# The Midterm Outcomes of Endovascular Therapy for Femoropopliteal Lesions via Drug-Coated Balloon, Directional Atherectomy and Bare Metal Stent Angioplasty

**DOI:** 10.31083/j.rcm2509331

**Published:** 2024-09-18

**Authors:** Yuhao Lin, Jianjun Quan, Jian Dong, Longlong Cong, Lin Yang

**Affiliations:** ^1^Department of Vascular Surgery, The First Affiliated Hospital of Xi’an Jiaotong University, 710061 Xi’an, Shaanxi, China; ^2^Department of Interventional Vascular Surgery, Hanzhong Central Hospital, 723000 Hanzhong, Shaanxi, China

**Keywords:** femoropopliteal lesion, endovascular therapy, directional atherectomy, drug-coated balloon, atherosclerosis, bare metal stent, angioplasty

## Abstract

**Background:**

This study investigated the midterm primary patency of drug-coated balloons (DCBs), directional atherectomy plus balloon angioplasty (DA), and bare metal stent (BMS) angioplasty for the treatment of femoropopliteal lesions.

**Methods:**

This two-center retrospective cohort study included 105 patients (110 limbs) undergoing DCB, DA, and BMS angioplasty—32 patients (34 limbs), 31 patients (32 limbs), and 42 patients (44 limbs), respectively. The demographic, baseline, and procedure data were collected, and the complications and midterm outcomes (patency, amputation-free survival, and clinically driven target lesion revascularization rates) were analyzed.

**Results:**

All three procedures achieved a 100% success rate. Significant improvements were noted in ankle brachial index, walking distance, and Rutherford classification at 30 days post-procedure (*p* < 0.001), with no differences or severe complications among the groups. The all-cause mortality rate during the follow-up period, was 5.5%, and amputation-free survival rates at 24 months were 97.0%, 90.6% and 90.9% in the DCB, DA, and BMS angioplasty groups, respectively. The primary patency rate for the DCB group (79.4%) exceeded those of the DA (56.2%) and BMS (52.2%) groups (*p* < 0.05), with no significant difference between the DA and BMS groups at 24 months. The secondary patency and clinically driven target lesion revascularization rates were similar among the three groups. A runoff number ≤1, Trans-Atlantic Intersociety Consensus (TASC) D, and severe calcification were found to be independent risk factors for primary patency.

**Conclusions:**

The DCB procedure demonstrated superior primary patency, compared to both BMS and DA procedures, in the treatment of femoropopliteal lesions.

## 1. Introduction

Femoropopliteal lesions (FPLs), characterized by stenosis or occlusion, are a common manifestation of peripheral artery disease, leading to severe limb ischemia, which can ultimately result in limb amputation or death [1, 2, 3, 4, 5]. While the classical surgical vein bypass remains the gold standard for treating FPLs, various endovascular procedures including bare metal stents (BMSs), drug-coated balloons (DCBs), and directional plaque atherectomy combined with balloon angioplasty (DA) have been shown to be effective and safe alternatives. Given the unique mechanical properties of the femoropopliteal artery, these interventions exhibit varied clinical outcomes [6, 7, 8, 9].

While, DCB angioplasty is the most common therapeutic approach for FPLs, BMS and DA combined with angioplasty are still important therapeutic procedures for complex FPLs (such as those with long-length occlusion and severe calcification) [7, 8, 9]. These procedures are frequently used in conjunction with DCB angioplasty. Furthermore, the selection of treatment modalities can be influenced by physicians’ preferences, patients’ economic status, and medical insurance policies, which vary significantly across different regions and hospitals [9, 10]. This variability poses substantial challenges to replicating clinical strategies in randomized controlled trials for complex FPLs. Therefore, clinical studies that investigate real-world applications are essential to assess the effectiveness and safety of these diverse procedures. In this study, we retrospectively analyzed the 2-year clinical outcomes of endovascular therapy for complex FPLs, focusing on factors affecting the patency rate.

## 2. Patients and Methods

The data that support the findings of this study are available from the corresponding author upon reasonable request.

### 2.1 Patients and Study Design

In this two-center retrospective cohort study, we included 105 patients (57 men) with FPLs treated between January 2020 and January 2021. Baseline data, procedural details, and follow-up outcomes were collected for each patient. Diagnostic evaluations were performed using pre-procedure ultrasound and computed tomography angiography (CTA). Treatment modalities varied among the patients: 32 underwent DCB angioplasty, 31 patients underwent DA plus balloon angioplasty, and 42 patients underwent BMS angioplasty (primary stenting). The inclusion criteria were as follows: (1) ≥40 years of age; (2) symptomatic with a Rutherford classification of 3–6; and (3) distal runoff vessels ≥1. The exclusion criteria were as follows: (1) history of previous endovascular therapy; (2) severe stenosis or occlusion of the aortoiliac artery; (3) acute arterial thrombosis; (4) cardioembolic disease; (5) inflammatory or immune vascular disease; (6) allergy to the contrast media; (7) hemorrhagic disease within the previous 6 months; (8) other contraindications to anesthesia or procedures; and (9) loss to follow-up. This study was approved by the Ethics Committee of the First Affiliated Hospital of Xi’an Jiaotong University and was conducted in accordance with the Declaration of Helsinki and all local regulatory requirements.

### 2.2 Endovascular Procedures

All patients received dual-antiplatelet therapy (aspirin 100 mg/day, clopidogrel 75 mg/day) at least 3 days before the endovascular procedure and for 3 months after the procedure. Afterward aspirin (100 mg/day) was used as maintenance therapy. Atorvastatin (20 mg/day) was administered as a lipid-lowering therapy during the entire treatment process.

The endovascular procedure was performed under local anesthesia with lidocaine, and the contralateral common femoral artery was used as the access site for retrograde puncture. A support catheter and guide wire were used to cross the target lesion in an antegrade direction after heparinization (80 U/kg). If the guide wire could not cross the lesion, retrograde puncture at the distal normal artery was performed to build the work wire, and then the procedure was carried out via the contralateral common femoral artery.

After accessing the lesion, plain balloon angioplasty was performed for artery lumen preparation. Bare metal stents (all stents were self-expanding stents) were placed in the patients in the BMS group. Post-dilation was performed when the recoil was >30%. In the DCB group, a drug-coated balloon (AcoArt Orchid, Beijing, China) was used after plain balloon angioplasty, and a bailout stent was used in cases of flow-limiting dissection or recoil >30%.

In the DA group, a TurboHawk atherectomy catheter (Medtronic, Minneapolis, MN, USA) was used to treat the target lesion in a proximal-to-distal direction under the guidance of the guide wire (without intravascular ultrasound), and the debulking speed and angle were controlled as previously reported. A distal protection filter (Spider FX; Medtronic, Minneapolis, MN, USA) was selectively used based on the plaque load. Depending on the fixation direction, the target lesion was divided into four quadrants for atherectomy, and the operator cleared the plaque debris from the collection tank in a timely manner to prevent debris from causing a distal embolism. After plaque removal, the target lesion was dilated with plain balloon angioplasty and then treated with a bare metal stent if the recurrence rate was >30% or if flow-limiting dissection occurred. After the endovascular therapy procedure, the access sites were closed using closure devices (Exseal, Cordis).

### 2.3 Study Endpoints and Definition

Procedure success was defined as residual stenosis <30% on the final angiography after angioplasty, and restenosis was defined as >50% based on angiography- or ultrasound examination-derived velocity parameters (peak systolic velocity ratio ≥2.4) [11]. Primary patency was defined as a target lesion without occlusion/stenosis (<50%) or performance of clinically driven target lesion revascularization (CD-TLR) during the follow-up period. Secondary patency was defined as patency maintained after secondary endovascular therapy in patients with restenosis/occlusion after primary therapy. Amputation-free survival was defined as survival without any amputation or death during the follow-up period. Clinically driven target lesion revascularization was defined as repeated endovascular therapy for revascularization due to symptom deterioration and restenosis ≥70% (CTA) [12, 13]. The primary outcome was primary patency, and the secondary outcomes were CD-TLR and the amputation-free survival rate after the procedure.

The degree of target vessel calcification was defined based on the previously reported peripheral academic research consortium calcification system; briefly, focal/mild calcification was defined as <180° (1 side of a vessel) and less than/greater than one-half of the total lesion length; moderate calcification was defined as >180° (both sides of a vessel at the same location) and less than one-half of the total lesion length; and severe calcification was defined as >180° (both sides of a vessel at the same location) and greater than one-half of the total lesion length [12].

### 2.4 Follow-up

The follow-up was completed at an outpatient or local hospital at 1, 6, 12 and 24 months after the procedure. The ankle brachial index (ABI), walking distance and Rutherford classification were recorded at 1 month. The primary and secondary endpoint events were recorded at 6, 12, and 24 months. The patency of the target lesion was assessed by ultrasound examination; CTA was used to re-evaluate the target lesion for patients with recurrent symptoms, and further revascularization was performed according to the patients’ symptoms and restenosis of the target lesion.

### 2.5 Statistical Analysis

All of the data were collected and organized using Microsoft Excel 2010 (Microsoft, Redmond, WA, USA), and SPSS 25.0 software (IBM Corp., Chicago, IL, USA) was used to perform the statistical analysis. The Kolmogorov‒Smirnov test assessed the distribution of the measurement data. Continuous data that conformed to a normal distribution, were expressed as mean and standard deviation (SD) and were analyzed using one-way analysis of variance (ANOVA). Categorical variables are presented as percentages and were evaluated using the chi-square test or Fisher’s exact test, as appropriate. Kaplan‒Meier survival analysis was used to calculate the primary patency, secondary patency, restenosis, CD-TLR and amputation-free survival rates. The risk factors for primary patency were determined by using univariate and multivariate Cox hazard regression analyses, and hazard ratios (HRs) and 95% confidence intervals (CIs) were calculated. *p* < 0.05 was considered statistically significant.

## 3. Results

### 3.1 Characteristics of the Baseline and Lesion Data

In this retrospective cohort study, 105 FPL patients were included, encompassing a total of 110 limbs. The distribution of interventions among the patients was as follows: 32 patients (34 limbs) underwent the DCB procedure, 31 patients (32 limbs) underwent the DA procedure, and 42 patients (44 limbs) underwent the BMS procedure (Table 1). Men comprised 56.3%, 48.4% and 57.1% of the three groups, respectively (*p* > 0.05). The demographic and baseline characteristics, including the distribution of gender across the groups, showed no significant differences (*p* > 0.05), indicating a well-balanced sample across the treatment modalities.

**Table 1.** **Baseline demographic and clinical characteristics of the study cohort**.

	DCB (n = 32)	DA (n = 31)	BMS (n = 42)	p*
Age (years)	69.76 ± 9.40	70.13 ± 9.27	69.41 ± 9.29	0.94
Male	18 (56.3)	15 (48.4)	24 (57.1)	0.73
BMI	23.02 ± 2.88	24.15 ± 2.79	23.18 ± 3.15	0.24
Smoking	20 (62.5)	14 (45.2)	23 (54.8)	0.38
Drinking	9 (28.1)	11 (35.5)	17 (40.5)	0.54
Hypertension	20 (62.5)	23 (74.2)	27 (64.3)	0.56
Diabetes	15 (46.9)	16 (51.6)	22 (52.4)	0.88
CAD	11 (34.4)	12 (38.7)	15 (35.7)	0.93
Stroke	9 (28.1)	7 (22.6)	14 (33.3)	0.60
Hyperlipidemia	14 (43.8)	16 (51.6)	18 (42.9)	0.73
H-HCY	10 (31.3)	7 (22.6)	10 (23.8)	0.68
COPD	3 (9.4)	2 (6.5)	8 (19.0)	0.22
CKD	4 (12.5)	3 (9.7)	6 (14.3)	0.83

DCB, drug-coated balloon; DA, directional atherectomy; BMS, bare metal stent; BMI, body mass index; CAD, coronary atherosclerotic disease; H-HCY, hyperhomocysteinemia; COPD, chronic obstructive pulmonary disease; CKD, chronic kidney disease. *p value, comparison among groups.

Total occlusion lesions accounted for 91.2%, 87.5% and 88.6% of the lesions in the DCB, DA, and BMS groups respectively, and the mean lesion lengths were 20.2 cm, 18.7 cm, and 20.8 cm, correspondingly. There were no significant differences in the baseline or target lesion characteristics among the three groups, indicating a consistent treatment approach across different modalities (Table 2, *p* > 0.05). This uniformity is crucial for comparing outcomes across treatment strategies effectively.

**Table 2.** **Comparison of target lesion characteristics by treatment group**.

		DCB (n = 34)	DA (n = 32)	BMS (n = 44)	p*
Left limb		17 (50.0)	15 (46.9)	24 (54.5)	0.79
Type of lesion					0.94
	Occlusion	31 (91.2)	28 (87.5)	39 (88.6)	
	Stenosis	3 (8.8)	4 (12.5)	5 (11.4)	
Location of lesion					0.95
	SFA	15 (44.1)	17 (53.1)	22 (50.0)	
	SFA+Pop1/2	11 (32.4)	9 (28.1)	14 (31.8)	
	SFA+Pop3	8 (23.5)	6 (18.8)	8 (18.2)	
Rutherford classification					0.72
	3	19 (55.9)	13 (40.6)	24 (54.5)	
	4	8 (23.5)	10 (31.3)	12 (27.3)	
	5	3 (8.8)	6 (18.8)	6 (13.6)	
	6	4 (11.8)	3 (9.4)	2 (4.5)	
TASC II classification					0.55
	B	12 (35.3)	11 (34.4)	10 (22.7)	
	C	10 (29.4)	6 (18.8)	13 (29.5)	
	D	12 (35.3)	15 (46.9)	21 (47.7)	
PARC calcification					0.35
	Mild	12 (35.3)	10 (31.3)	20 (45.5)	
	Moderate	9 (26.5)	14 (43.8)	14 (31.8)	
	Severe	13 (38.2)	8 (25.0)	10 (22.7)	
Pre-procedure ABI		0.37 ± 0.25	0.35 ± 0.25	0.36 ± 0.23	0.91
Pre-procedure claudication (m)		140.00 ± 93.58	158.75 ± 114.38	148.41 ± 101.64	0.76
Length of lesion (cm)		20.21 ± 11.25	18.72 ± 9.80	21.80 ± 8.63	0.66
Proximal diameter (cm)		4.79 ± 0.39	4.78 ± 0.35	4.86 ± 0.35	0.55
Distal diameter (cm)		4.26 ± 0.36	4.23 ± 0.36	4.27 ± 0.30	0.85

DCB, drug-coated balloon; DA, directional atherectomy; BMS, bare metal stent; SFA, superficial femoral artery; Pop, popliteal artery; TASC, Trans-Atlantic Intersociety Consensus; PARC, Peripheral Academic Research Consortium calcification system; ABI, ankle brachial index; m, meter; cm, centimeter. *p value, comparison among groups.

### 3.2 Procedural Parameters and 30 d Outcomes

**Fig. 1.**
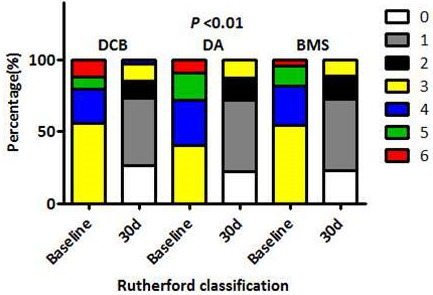
**Improvements in Rutherford classification across treatment groups at 30 days**. The Rutherford classifications in the DCB, DA, and BMS groups at 30 days post-procedure. Significant improvements were noted in all groups when compared to baseline (*p* < 0.01), with no significant differences detected between the groups (*p* > 0.05). DCB, drug-coated balloon; DA, directional atherectomy; BMS, bare metal stent.

Retrograde puncture was employed in 23.5%, 21.8%, and 11.3% of patients in the DCB, DA, and BMS groups, respectively, indicating its varied application across treatment modalities (Table 3, *p* > 0.05). Bailout stents were implanted in 7 limbs (20.6%) and 5 limbs (15.6%) in the DCB and DA groups, respectively. The incidences of flow-limiting dissection and recoil were similar among all groups (*p* > 0.05). No significant differences were detected in the final diameter of the target lesion or the incidence of access complications, and no severe complications, such as acute thrombosis, stroke, myocardial infarction or death, occurred. In the DA group, while no distal protection devices were used, one patient experienced distal embolism (3.1%), which was successfully treated with complete recanalization after catheter aspiration. At 30 days after the procedure, significant improvements were noted in both the ABI and walking distances, alongside a notable decrease in the Rutherford classification compared to pre-procedure values (*p* < 0.001), demonstrating the procedural efficacy across all groups (Fig. 1, *p* < 0.01).

**Table 3.** **Procedural parameters and 30-day clinical outcomes**.

		DCB (n = 34)	DA (n = 32)	BMS (n = 44)	p*
Antegrade through		26 (76.5)	25 (78.2)	39 (88.6)	0.31
Retrograde through		8 (23.5)	7 (21.8)	5 (11.3)	0.31
Pre-dilation balloon		34 (100)	32 (100)	44 (100)	
	2 mm	8 (23.5)	8 (25.0)	6 (13.6)	0.39
	3 mm	18 (52.9)	16 (50.0)	26 (59.1)	0.72
	4 mm	32 (94.1)	30 (93.8)	41 (93.2)	0.98
	5 mm	26 (76.5)	27 (84.4)	36 (81.8)	0.70
	6 mm	3 (8.8)	4 (12.5)	2 (4.5)	0.45
Post-dilation		3 (8.8)	7 (21.9)	9 (20.5)	0.28
Flow-limiting dissection		5 (14.7)	4 (12.5)	8 (18.2)	0.49
Recoil		2 (5.9)	1 (3.1)	5 (11.4)	0.38
Bailout stent		7 (20.6)	5 (15.6)		0.13**
Stent number		7	13	50	
Final diameter (mm)		5.29 ± 0.46	5.34 ± 0.48	5.39 ± 0.49	0.65
Distal filter		0	6 (18.75)	0	
Access complications		1 (2.9)	2 (6.3)	2 (4.5)	0.85
	Hematoma	1	1	1	
	Pseudoaneurysm	0	0	1	
	Bleeding	0	1	0	
Post-procedure ABI		0.84 ± 0.14	0.82 ± 0.15	0.82 ± 0.12	0.89
Post-procedure claudication		708.82 ± 440.64	765.63 ± 571.14	879.55 ± 577.72	0.36
Post-procedure Rutherford					0.95
	0	9 (26.4)	7 (21.9)	10 (22.7)	
	1	16 (47.1)	16 (50.0)	22 (50.0)	
	2	4 (11.8)	5 (15.6)	7 (15.9)	
	3	4 (11.8)	4 (12.5)	5 (11.4)	
	4	1 (2.9)	0 (0)	0 (0)	

DCB, drug-coated balloon; DA, directional atherectomy; BMS, bare metal stent; ABI, ankle brachial index. *p value, comparison among groups; **p value, comparison between the DCB and DA groups.

### 3.3 Follow-up Outcomes

**Fig. 2.**
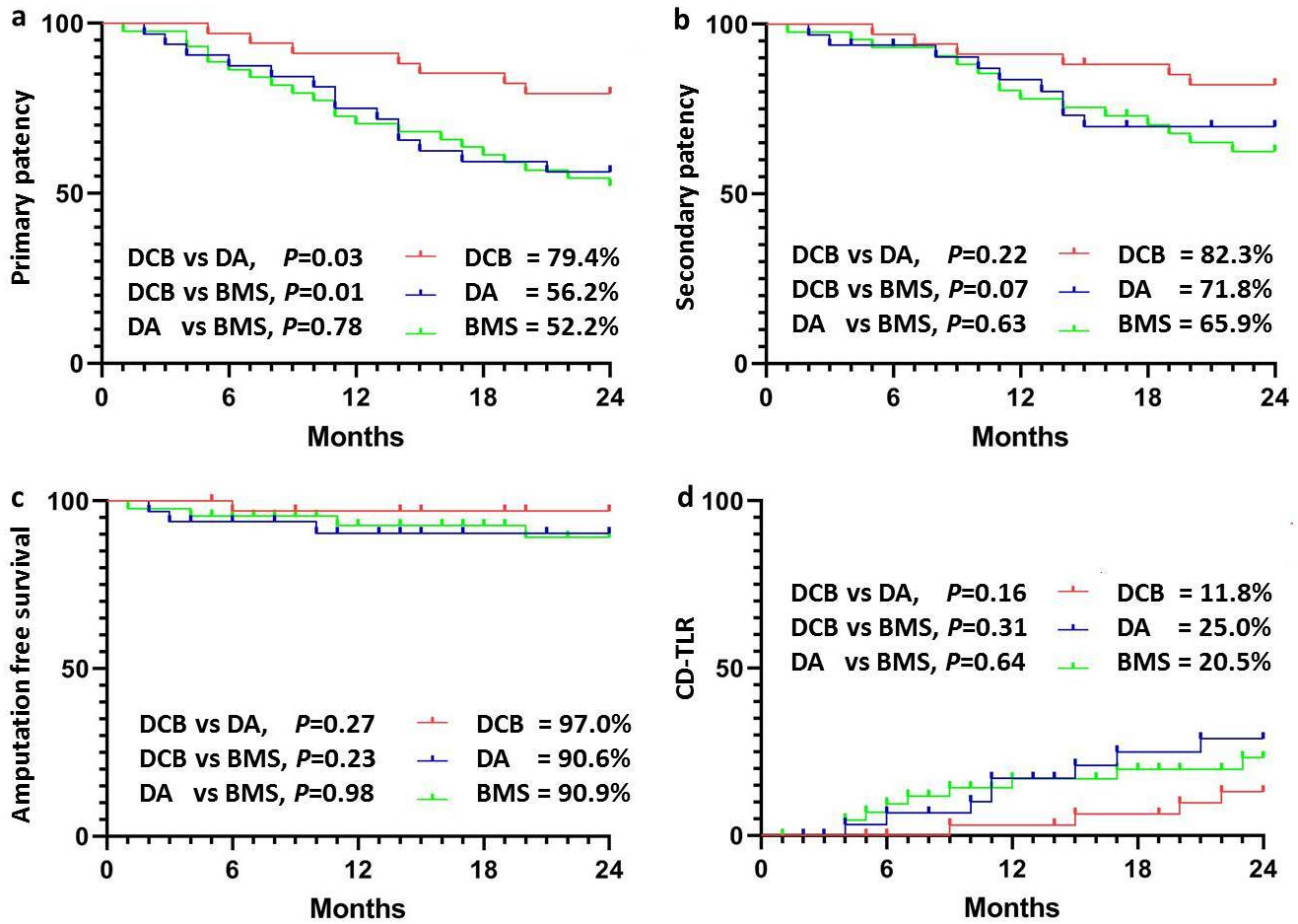
**Comparative analysis of follow-up outcomes across treatment groups**. Fig. 2 illustrates the primary and secondary patency rates, amputation-free survival, and clinically driven target lesion revascularization (CD-TLR) rates at 24 months post-procedure. (a) shows that the DCB group had a significantly higher primary patency rate compared to the DA and BMS groups (*p* < 0.05). (b–d) show no significant differences in secondary patency, amputation-free survival, and CD-TLR rates among the groups (*p* > 0.05). CD-TLR, clinically driven target lesion revascularization; DCB, drug-coated balloon; DA, directional atherectomy; BMS, bare metal stent.

During the follow-up period, the rates of loss to follow-up were 6.7% (7) at 6 months, 10.5% (4) at 12 months, and 17.1% (7) at 24 months. The overall mortality rate was 5.5%, including occurrences of myocardial infarction (three patients), stroke (one patient), and pulmonary infection (two patients). The primary patency rates at 24 months were 79.4%, 56.2% and 52.2% in the DCB, DA and BMS groups, respectively, according to Kaplan‒Meier analysis (Fig. 2a). The primary patency rate was greater in the DCB group than in the DA and BMS groups (*p* < 0.05), and no difference was detected between the DA and BMS groups (*p* > 0.05). In contrast, no difference in the secondary patency rate was detected among the DCB (82.3%), DA (71.8%) and BMS groups (Fig. 2b, 65.9%, *p* > 0.05). Three patients underwent planned amputation after the procedure (2.7%), and there were no differences in the amputation-free survival rates among the DCB, DA and BMS groups (Fig. 2c, *p* > 0.05). In the follow-up period, repeated endovascular therapy was required for four patients (11.8%) in the DCB group, eight patients (25.0%) in the DA group, and nine patients (20.5%) in the BMS group. No significant differences in CD-TLR rates were observed among the groups (Fig. 2d, *p* > 0.05).

### 3.4 Risk Factors for Primary Patency

In this study, CAD, lesion length ≥20 cm, Trans-Atlantic Intersociety Consensus (TASC) D classification, DCBs, and severe calcification were identified as risk factors for primary patency according to univariate analysis (Table 4). Additionally, multivariate Cox regression analysis highlighted more than one runoff vessel (hazard ratio [HR] = 2.24, 95% confidence interval [CI]: 1.20–4.18, *p* < 0.05), TASC D (HR = 4.30, 95% CI: 1.07–17.19, *p* = 0.04), and severe Peripheral Academic Research Consortium calcification system (PARC) calcification (HR = 2.45, 95% CI: 1.09–5.50, *p* = 0.03) as independent risk factors for primary patency, indicating a significantly higher risk of patency loss under these conditions.

**Table 4.** **Risk factors for primary patency: univariate and multivariate analysis**.

	Univariate			Multivariate		
	HR	95% CI	*p*	HR	95% CI	*p*
Male sex	1.43	0.78–2.63	0.25			
H-HCY	1.44	0.76–2.74	0.27			
CAD	2.12	1.16–3.89	0.02			
Stroke	1.64	0.89–3.05	0.16			
Lesion length ≥20 cm	1.95	1.04–3.64	0.04			
Involvement in P1-3	1.97	0.93–4.18	0.08			
TASC D	2.65	1.24–5.64	0.01	4.30	1.07–17.19	0.04
Retrograde puncture	0.58	0.29–1.16	0.12			
DA	0.76	0.40–1.44	0.40			
DCB	0.37	0.17–0.84	0.02			
BMS	1.93	1.02–3.67	0.04			
Runoff vessels ≤1	0.68	0.37–1.24	0.21	2.24	1.20–4.18	0.01
Severe calcification	2.29	1.05–4.98	0.04	2.45	1.09–5.50	0.03
Proximal diameter ≥4.8 mm	1.62	0.86–3.05	0.13			

HR, hazard ratio; CI, confidence interval; H-HCY, hyperhomocysteinemia; CAD, coronary atherosclerotic disease; TASC, Trans-Atlantic Intersociety Consensus; DA, directional atherectomy; DCB, drug-coated balloon; BMS, bare metal stent.

## 4. Discussion

BMS implantation following balloon angioplasty remains a prevalent method for treating FPLs. However, patients undergoing this treatment are prone to long-term lumen loss and a high restenosis rate, which are key factors affecting long-term patency [1, 4, 7]. Matsumi* et al*. [14] reported that the primary patency rate was 87.7% at one year after the BMS procedure in 319 femoropopliteal artery lesions. Our study found that the 2-year primary patency rate of the endovascular treatments in patients with complex FPLs was 52.2%, while the primary patency rate of the DCB procedure was 79.4% (*p* < 0.05). The primary patency rate of the BMS procedure continuously decreased over time, and potential risk factors, such as TASC C/D and long lesions appeared to influence these outcomes. A previous report suggested that the ten-year patency rate could be as low as 29.1% [15]. Patients who underwent the BMS procedure had a lower primary patency rate than did those who underwent the DCB procedure in our study; however, the amputation-free survival rate and CD-TLR rate were similar.

Our findings are consistent with recent reports suggesting superior patency and lower revascularization rates for the DCB procedure compared to BMS [16, 17]. Consequently, the DCB procedure emerged as a more effective first-line therapy for endovascular treatment of FPLs. However, the DCB procedure, being a form of balloon angioplasty, may not always achieve optimal clinical outcomes for complex FPLs, necessitating adjunctive procedures such as provisional BMS and atherectomy to achieve better clinical results [18].

In DA, physical or mechanical methods are used to remove thrombi or plaques, effectively enlarging the lumen of the targeted vessels [8]. This process significantly reduces the incidence of flow-limiting dissection during subsequent balloon angioplasty [8]. Enhancing the lumen diameter not only facilitates drug delivery through the vessel walls but also reduces the necessity for bailout stent deployment [8]. These improvements contribute to the long-term patency of treated lesions [8]. The DEFINITIVE AR study results indicate that the incidence of flow-limiting dissection in the DA+DCB group (2%) was significantly lower compared to the group treated with DCB alone (19%) [19]. Additional studies confirmed that the clinical outcomes of DA combined with DCBs were superior to those of DCBs alone [19, 20]. However, previous study has suggested that patients who underwent DA alone had greater reintervention rates than patients who did not undergo the DA procedure [21].

Our findings align with these observations; DA combined with plain angioplasty showed no additional clinical benefits, and the incidence of flow-limiting dissection in the DA group was similar to that in the DCB and BMS groups. Moreover, the primary patency rate in the DA group was lower than in the DCB group, underscoring limited benefits of DA alone. Recent study corroborates these findings, noting comparable outcomes between DA alone and plain balloon angioplasty [22]. Therefore, DA combined with DCBs may achieve better clinical outcomes, which is consistent with existing research [23]. Furthermore, the clinical benefit of other atherectomy methods, such as rotational or laser plus DCB, is affirmed in the treatment of femoropopliteal artery and below-the knee lesions [8, 24, 25, 26], suggesting atherectomy plus DCB should be the routine practice for most endovascular specialists. However, more evidence need to be verified in the treatment of the below-the-knee lesions.

For complex lesions, such as those with TASC D, severe calcification, long-segment occlusion, and diabetic foot lesions, the current procedures still exhibit high restenosis rates [8]. Therefore, it is important to evaluate the risk factors that affect the results of endovascular therapy for FPLs before surgery. Iida* et al*. [27] reported that poor post-procedure patency of below-the-knee vessels is an independent risk factor for the occurrence of restenosis following various types of revascularizations. Our results demonstrated that in patients with fewer than one runoff vessel below the knee, TASC D classification and severe calcification were independent risk factors for restenosis, aligning with previous reports [28, 29]. Severe calcification, TASC D classification, and long-segment occlusion, which are the main risk factors for primary patency, occur in complex FPLs, and influence the results of balloon angioplasty, stent implantation and drug absorption of DCBs [8, 29]. Moreover, other risk factors also affect the patency of FPLs. Therefore, it is necessary to provide precise therapeutic options according to the characteristics of the target lesion and to select therapeutic strategies such as DA, DCB and stent angioplasty in clinical practice.

## 5. Limitations

Although our study generated useful confirmatory conclusions, it also has several limitations. First, this was a small sample size two-center retrospective cohort study, which may limit the generalizability of our conclusions. Future studies should aim to verify our findings through randomized clinical trials with large sample sizes. Second, the DA group did not include those who underwent DCB angioplasty due to the prohibitive cost and lack of medical insurance coverage in our region, which may have introduced bias into our results. Third, our study did not include newer nitinol stent devices, which could affect the generalizability of the conclusions. Finally, all procedures were performed without intravascular ultrasound guidance, and the lumens were not clearly tracked, which affects the accuracy and objectivity of the conclusions.

## 6. Conclusions

Our results suggest that the primary patency rate was greater for the DCB procedure than for the DA and BMS procedures at the 24-month follow-up. Therefore, the DCB procedure might be the optimal first line treatment for FPLs.

## Availability of Data and Materials

The data of the study in this article will be made available by the authors upon reasonable reasons.
